# Automated joint segmentation enables quantitative molecular imaging of degeneration with [¹⁸F]FDG and [¹⁸F]NaF PET/CT

**DOI:** 10.1007/s00259-026-08139-z

**Published:** 2026-08-21

**Authors:** William K. Lee, Shiv Patil, Darshil H. Patel, Zhuorui Li, Ondrej Fanta, Om H. Gandhi, Thomas J. Werner, Poul F. Høilund-Carlsen, Abass Alavi

**Affiliations:** 1 Department of Radiology, Hospital of the University of Pennsylvania, Philadelphia, 19104 PA USA; 2 Drexel University College of Medicine, Philadelphia, PA USA; 3 Sidney Kimmel Medical College, Thomas Jefferson University, Philadelphia, PA USA; 4 First Faculty of Medicine, Charles University, Prague, Czechia Czechia; 5 Department of Nuclear Medicine, Odense University Hospital, Odense, Denmark; 6 Department of Clinical Research, University of Southern Denmark, Odense, Denmark

**Keywords:** Automated segmentation, Weight-bearing joints, Intervertebral disc, Osteoarthritis, Body mass index, Aging

## Abstract

**Purpose:**

Manual quantification of joint metabolism and inflammation with PET is time-consuming and difficult to standardize due to ambiguous joint boundaries. We aimed to develop an automatic joint segmentation method that efficiently and reliably quantifies activity in weight-bearing joints using [^18^F]fluorodeoxyglucose (FDG) and sodium [^18^F]fluoride (NaF) PET/computed tomography (PET/CT).

**Methods:**

Segmentations were generated from CT scans using the TotalSegmentator model. From these, 30 weight-bearing joints were segmented by identifying the region between adjacent bones within a set distance threshold. Scans of healthy volunteers were analyzed (75 with FDG, 80 with NaF). Mean standardized uptake value (SUVmean) was calculated for each joint region, and Pearson correlation analysis was used to evaluate relationships with age and body mass index (BMI). Benjamini–Hochberg false discovery rate (FDR) correction was applied within each tracer–covariate family.

**Results:**

The automated approach reduced segmentation time from 30 to ~ 1.5 min per scan. Joint ROI volumes generated independently from the two PET/CT sessions of the same participant were highly concordant (median *r* = 0.98, median intraclass correlation coefficient 0.94). After false discovery rate correction, NaF uptake was positively correlated with BMI in the thoracic (T3-T6, T7-T8, T9-T11) and lumbosacral (L5-S1) intervertebral discs (IVDs) and in the sacroiliac (SI), hip and knee joints, and positively with age in the cervical IVDs (C1-C2, C3-C7), with negative age correlations at T4-T5, T6-T7, L1-L2 and in both SI joints (all q < 0.05). FDG uptake was positively correlated with BMI in the IVDs from C1-T6 (other than T1-T2) and T9-S1, in both SI joints, the left hip and both knees, and with age in both knees, and negatively with age at T2-T3 and in both SI joints (all q < 0.05). Associations meeting *p* < 0.05 but not q < 0.05 are identified in Tables 2, 3, 4 and 5. The positive cervical NaF-age associations arise in the only joints where SUVmean was appreciably correlated with ROI size and are therefore reported as provisional.

**Conclusion:**

Our segmentation approach rapidly quantifies joint metabolism and revealed significant associations between PET tracer uptake, BMI, and age. It offers a standardized framework for future PET/CT joint analysis.

**Clinical trial registration:**

Cardiovascular Molecular Calcification Assessed by 18 F-NaF PET/CT (CAMONA), ClinicalTrials.gov NCT01724749. https://clinicaltrials.gov/study/NCT01724749.

**Supplementary Information:**

The online version contains supplementary material available at https://doi.org/10.1007/s00259-026-08139-z.

## Introduction

 Degenerative joint diseases are a leading cause of arthritis and disability characterized by the progressive breakdown of extracellular matrix in articular cartilage within joint spaces. Although the exact mechanism driving increased prevalence of degenerative changes in aging is not yet completely understood, several age-related processes have been implicated including cellular senescence, inflammation, oxidative stress, and limited regenerative capacity [1]. In addition, mechanical factors including weight-bearing load, biomechanical patterns of movement, and trauma have been strongly associated with degenerative changes, particularly osteoarthritis (OA) [2]. Overall, pain, physical disability, and an associated reduction in quality of life secondary to age-related degenerative joint changes imposes a significant burden on the aging population.

Positron Emission Tomography (PET) is a functional imaging modality that has been used to study pathophysiology of arthropathies. Besides oncological applications, [^18^F]fluorodeoxyglucose (FDG) PET/computed tomography (CT) has been widely used to study inflammatory disorders by tracking the glycolytic activity of inflammatory cells. Sodium [^18^F]fluoride (NaF) is another PET radiotracer suitable for tracking osteoblastic activities and bone turnover by binding to fluorapatite that is incorporated into the crystalline matrix of bone [3]. Several studies have utilized both FDG and NaF tracers in studying musculoskeletal disorders including OA, rheumatoid arthritis, and osteoporosis [4–12].

Currently, PET/CT radiological studies rely on manual or semi-automatic segmentation methods with thresholding techniques to define regions of interest (ROIs). This approach can be labor intensive and error-prone, particularly in the case of spinal joint segmentation due to the number of joints involved and variable anatomy at the time of scan. Selecting spherical ROIs instead of analyzing the whole organ or space is more convenient for researchers, but this approach can further introduce inconsistency in ROI definition and interobserver variability, reducing reproducibility of study results. With recent advances in the use of artificial intelligence (AI) in radiology and molecular imaging, efforts are underway to address the limitations of manual and semi-automatic segmentation methods. In this study, we introduce a novel segmentation method to achieve automatic simultaneous definition of several weight-bearing joints on PET/CT using an available AI-segmentation model, TotalSegmentator, based on the nnU-Net architecture [13–15]. We aim to validate this approach by correlating FDG and NaF uptake in weight-bearing joints with age and body mass index (BMI), two of the most significant risk factors for age-related joint degeneration [16].

## Methods

### Study design

This retrospective cross-sectional study evaluated metabolic activity in weight-bearing joints, including the intervertebral discs (IVDs), hip, sacroiliac (SI), and knee joints, using FDG and NaF PET/CT imaging. The data were obtained from the prospective Cardiovascular Molecular Calcification Assessed by NaF PET/CT (CAMONA) study, conducted at Odense University Hospital between 2012 and 2014. The CAMONA cohort included 89 healthy volunteers and 50 patients with angina pectoris and elevated cardiovascular risk, all of whom underwent both FDG and NaF PET/CT scans.

Angina pectoris patients were recruited from the Department of Cardiology, whereas healthy volunteers were recruited from the blood bank at Odense University Hospital or through local advertisements. Exclusion criteria for healthy volunteers consisted of the following: history of malignancy, immunodeficiency, autoimmune disorders, substance abuse, major skeletal deformities, or cardiovascular diseases. For this study, participants were selected based on scan quality and compatibility with TotalSegmentator. Four healthy participants were excluded from FDG scans due to scan artifacts but were included for NaF imaging; the remaining shortfall relative to the 89 eligible volunteers reflects sessions for which a usable scan of the relevant tracer was not available. One further FDG scan returned non-physiological SUVs at every joint, indicating a failure of SUV normalization, and was excluded from all analyses, leaving 75 FDG and 80 NaF scans in the correlation analyses. The final study population included individuals aged 21 to 75 years. Volunteers were not screened radiographically or clinically for degenerative joint disease, and neither symptomatic osteoarthritis nor degenerative disc disease was an exclusion criterion. The cohort therefore is an ambulatory population free of malignancy, immunodeficiency, autoimmune disease, substance abuse, major skeletal deformity and cardiovascular disease, and it was not verified to be free of structural joint pathology. Subclinical and asymptomatic degenerative change would be expected at the prevalence reported for a general population of comparable age and BMI.

### PET/CT protocol

The imaging protocol used in this study was previously described [17, 18]. FDG and NaF PET/CT were performed on integrated PET/CT systems (Discovery 690/710, STE, VCT and RX models; GE Healthcare, Chicago, Illinois, USA) at Odense University Hospital, Denmark.

For FDG PET/CT imaging, the procedure began 180 min after an intravenous injection of approximately 4.0 MBq/kg of FDG, following an overnight fast of at least 8 h. Blood glucose levels were assessed before injection to ensure they were below 8 mmol/L. NaF PET/CT was performed 90 min following intravenous administration of NaF at a dose of 2.2 MBq/kg.

The estimated effective radiation doses for patients were 6.5 mSv for FDG PET/CT and 6.0 mSv for NaF PET/CT. After the injections, participants rested in a quiet and warm environment. Each scan was acquired over a duration of 2.5 min per bed position.

PET images were corrected for random coincidences, scatter, attenuation, and anatomical alignment using transmission maps generated from a 64-slice CT scan. The CT parameters were set to 120 kV, 200 mA, with a collimation of 16 × 2.5 mm and a rotation time of 0.5 s.

### Image analysis

**Fig. 1:**
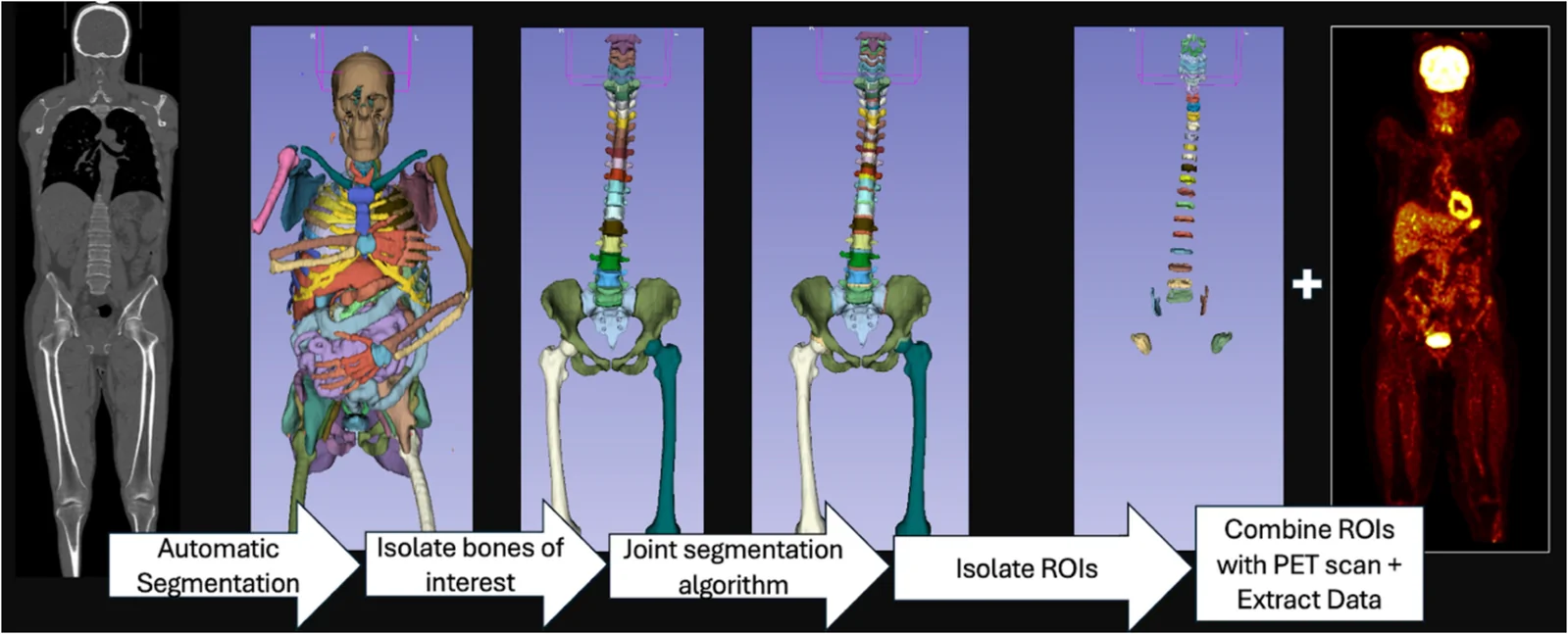
Segmentation and statistical analysis workflow. TotalSegmentator is used to generate an initial segmentation from the CT scan. Bones surrounding the joints of interest are then isolated, followed by application of a joint isolation algorithm to define regions of interest (ROIs). These ROIs are overlaid onto the corresponding PET scan to calculate the SUV_mean_ for each joint. CT indicates computed tomography; PET, positron emission tomography; SUV_mean_, mean standardized uptake value; ROI, region of interest.

**Fig. 2:**
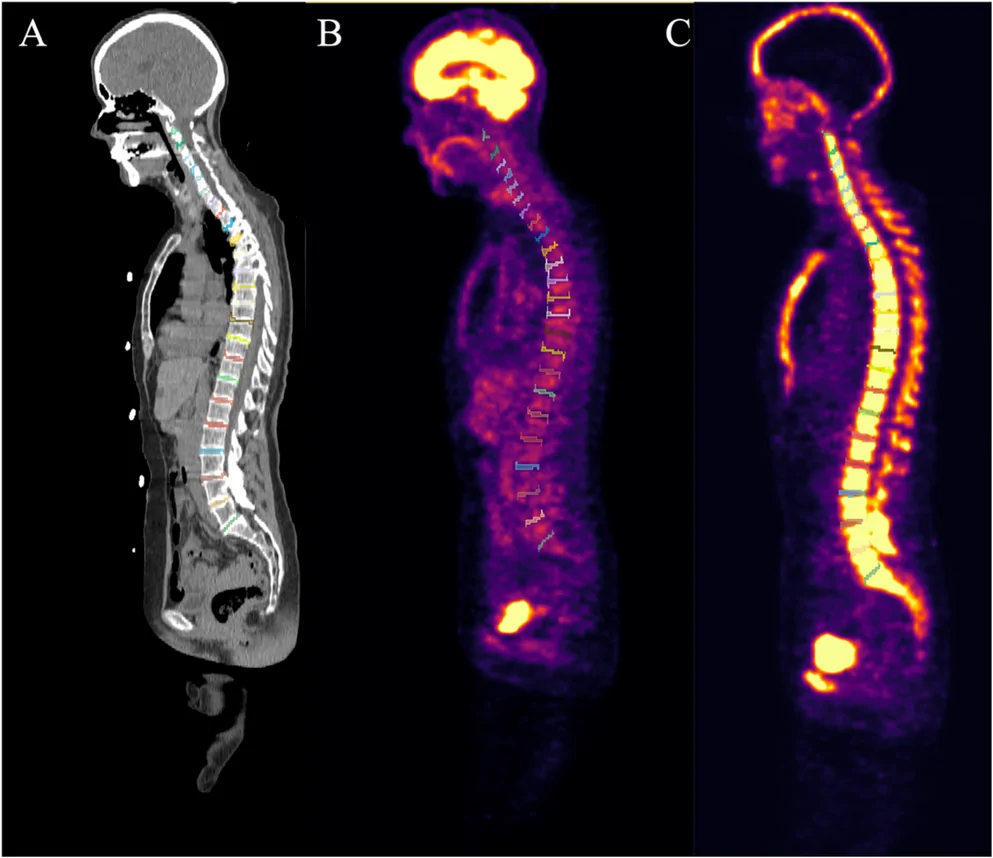
Sagittal views of a 62-year-old female patient with vertebral joint ROI overlays approximating the intervertebral disks. (**A**) CT scan showing anatomical reference of the spine. (**B**) ¹⁸F-FDG PET scan highlighting metabolic activity in the vertebral joints. (**C**) ¹⁸F-NaF PET scan demonstrating bone turnover and remodeling within the same ROIs

**Fig. 3:**
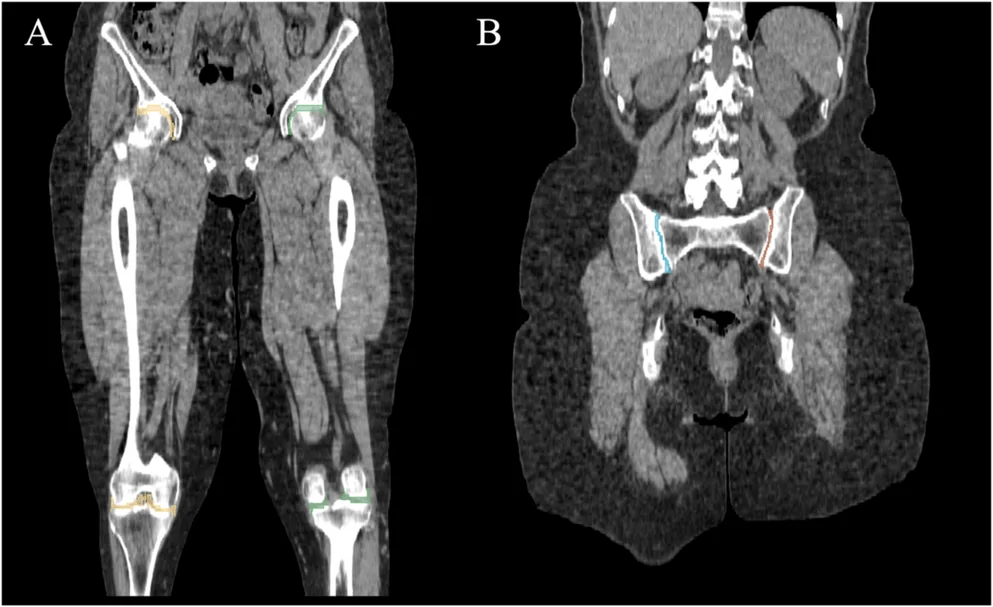
Coronal CT scans of a 62-year-old female patient with (**A**) hip and knee joint ROIs and (**B**) SI joint ROIs displayed. ROIs are shown as overlays on the anatomical images. CT indicates computed tomography; SI, sacroiliac; ROI, region of interest

PET standardized uptake values (SUVs) were calculated using CT-based segmentations that were created using an automatic segmentation method based on the publicly available TotalSegmentator model. To create the ROIs around the joints, the CT scans corresponding to each patient’s scan were first segmented using the “total” subtask available under the TotalSegmentator model. This segmentation model would output a segmentation mask with 117 different classes. Using this segmentation mask, we isolated the bone classes around our joints of interest. For example, to isolate the left knee joint, we extracted the left femur and tibia segmentations. Using these isolated bone segmentations, we ran our algorithm, which defined joint ROIs as being the set of points between two adjacent bones that were within a certain distance threshold (Fig. 1). For the IVDs, we defined this as being 2 voxels, while all the remaining joints were defined as 3 voxels. These thresholds are modifiable depending on the joint of interest, but we found that these values most accurately encapsulated the joint in our testing. Because the thresholds are expressed in voxels and not in millimeters, the physical dimensions of the resulting ROI scale with the reconstructed voxel size of the CT series, and the consequences of this are addressed in the Limitations. Once these ROIs were generated around our joints of interest, they were overlaid onto the corresponding PET scan and the mean standardized uptake value (SUVmean) was calculated as being the average SUV value of the voxels inside the ROIs (Figs. 2 and 3).

Thin colored overlays represent sagittal cross-sections of 3D volumetric ROIs generated around each intervertebral joint using a distance-based approach. Each ROI spans the entire intervertebral space. ROI indicates region of interest; CT, computed tomography; FDG, fluorodeoxyglucose; NaF, sodium fluoride; PET, positron emission tomography.

### Computational environment

All processing was performed locally on a single desktop workstation equipped with an NVIDIA GeForce RTX 3080 GPU (10 GB VRAM); no institutional cluster, cloud service or dedicated server hardware was used. TotalSegmentator was run through its Python interface using the default settings of the “total” task, and the joint-isolation algorithm, ROI generation and SUV extraction were implemented in Python. Under this configuration, generation of all 30 joint ROIs and the associated SUVmean values required an average of approximately 1.5 min per scan.

### Verification

The accuracy of our method depends on the accuracy of the underlying bone segmentations, because an inaccurate TotalSegmentator output means that the distance-based method will not function correctly. We ensured accuracy by applying an “appearance threshold”, which filters out any bone segmentations and calculated joint ROIs that are less than 50 voxels. We found that these values filtered out any floating mislabeled segmentations that would introduce errors into our statistical calculations. Notably, joints that extended beyond the scan field of view, like the elbows, were excluded by this appearance threshold.

**Fig. 4:**
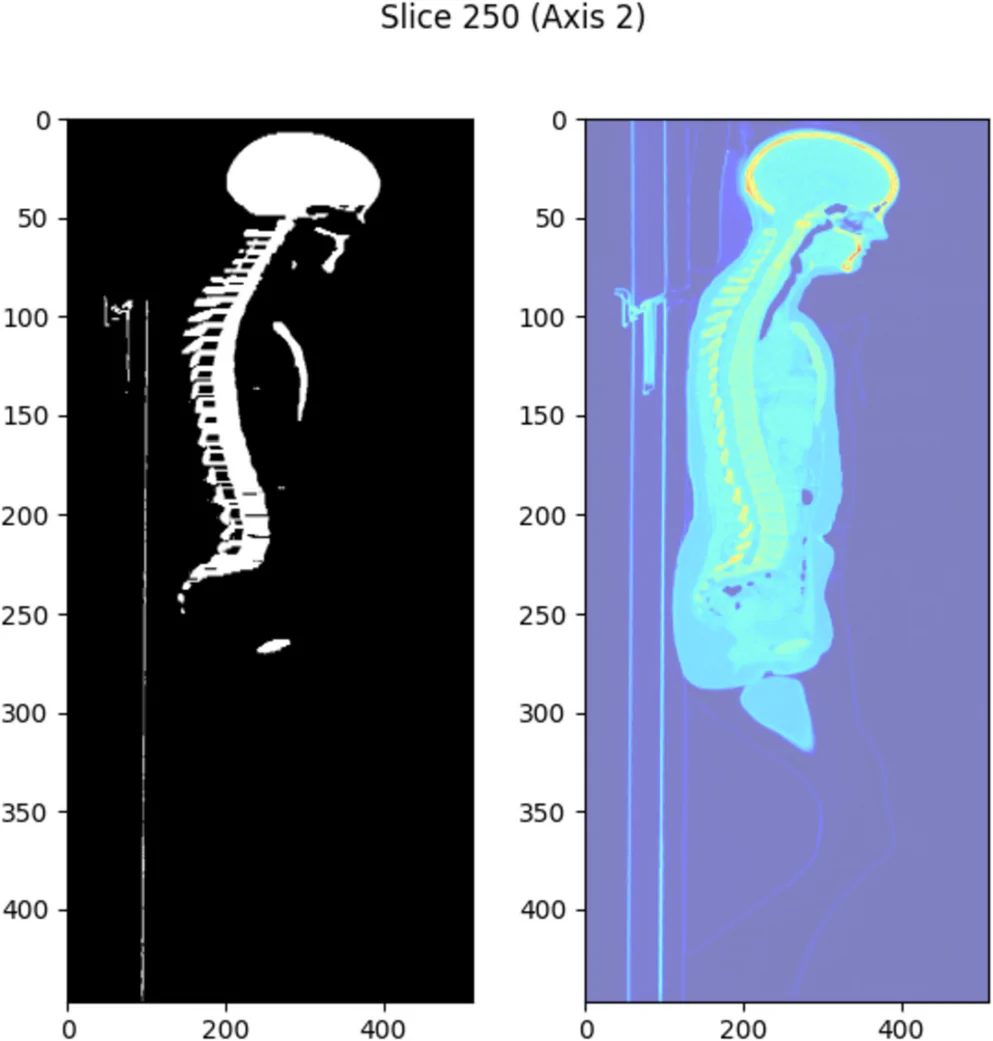
Left: Bone segmentation mask generated through CT scan HU thresholding and denoising. Right: Corresponding CT scan used for masking. CT indicates computed tomography; HU, Hounsfield Units

**Fig. 5:**
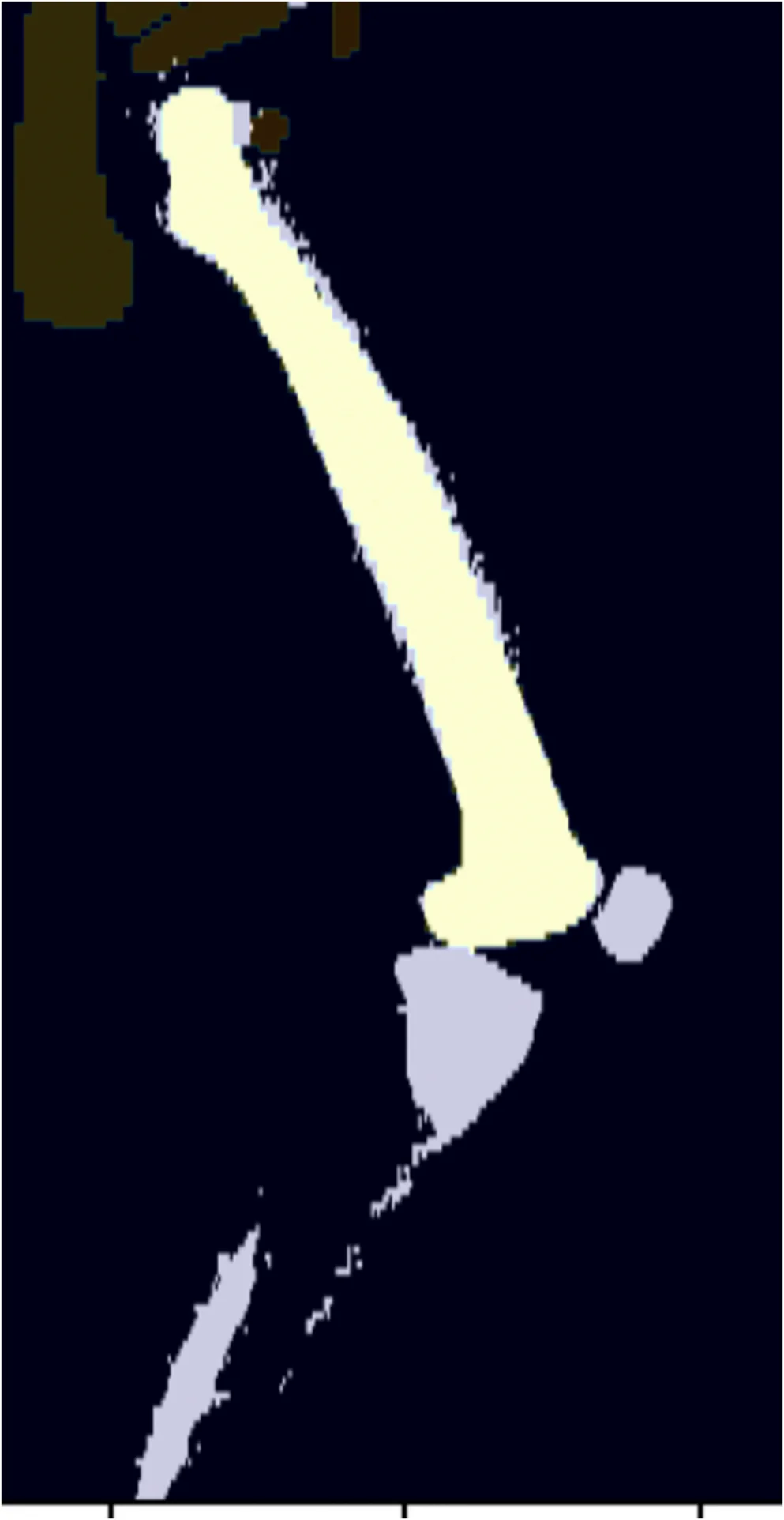
Overlap of femoral bone segmentations on CT used for accuracy assessment. The blue region is the bone mask obtained by Hounsfield unit (HU) thresholding, which served as the reference, and the yellow region is the segmentation generated by TotalSegmentator, which was the output under evaluation. Overlapping regions indicate agreement between methods, while non-overlapping regions highlight areas of discrepancy. Accuracy was quantified as a one-sided overlap (containment index), defined as the proportion of the TotalSegmentator segmentation contained within the HU-derived bone mask, as described in the Methods. Other structures shown in this figure (fibula, tibia, patella) can be ignored as they were not involved in segmentation analysis

Finally, to verify the TotalSegmentator segmentation model bone segmentations, we took each patient’s CT scan and applied a Hounsfield unit (HU) threshold mask of 200 HU to isolate both cortical and trabecular bone in the CT scan. A denoising step was then applied to filter out noise in the form of small, high-HU artifacts (Fig. 4). We used this HU-thresholded image as the reference for the structures of interest. To evaluate segmentation accuracy, we calculated a one-sided overlap, defined as the number of overlapping voxels between the TotalSegmentator segmentation and the HU-derived bone mask, divided by the total number of voxels in the TotalSegmentator segmentation. A value of 1.0 indicates that the TotalSegmentator segmentation is fully contained within the corresponding HU mask. We computed two separate accuracy values for each radiotracer: one for the vertebral bones (assessing vertebral joint segmentations) and one for the remaining bones (e.g. femur, hip, sacrum) involved in the hip, knee and SI joints (Fig. 5). Our FDG scans had an average accuracy of 0.97 for the vertebrae and 0.95 for the hip, sacrum, and femur. For NaF scans, the average accuracy was 0.98 for vertebrae and 0.96 for the other bones. Unlike manual segmentation, which requires repeated operator decisions, the method is fully deterministic and produces an identical segmentation from an identical input scan. This one-sided overlap is a containment index that quantifies the proportion of each model-derived bone segmentation that falls within an independently derived, intensity-based bone mask, and it is by construction insensitive to voxels present in the HU-derived mask but absent from the model output. Expert manual delineations of the joint spaces themselves were not available for this retrospective cohort, so DSC and Hausdorff distance against a manually traced reference standard could not be computed; this is addressed in the limitations.

### Reproducibility and sensitivity analyses

Every participant underwent two PET/CT sessions on different days, one with FDG and one with NaF, each with its own low-dose CT acquisition and independent positioning. This allowed the stability of the ROI-generation step to be assessed without a dedicated scan–rescan acquisition. For each participant with both sessions available, the ROI volume obtained for a given joint from the FDG-session CT was compared with the volume obtained from the NaF-session CT, so that the two volumes came from independent runs of the pipeline on independently acquired images. The two sessions were not always reconstructed onto the same voxel grid, and participants whose paired ROI volumes differed by a consistent multiplicative factor across every joint, which indicates a difference in reconstructed voxel size and not in segmentation, were identified from the distribution of the per-participant median volume ratio and were excluded from this comparison. Agreement was summarized by the Pearson correlation coefficient, the intraclass correlation coefficient, the within-subject coefficient of variation and the median systematic difference, each computed per joint and summarized across joints. Two further sensitivity analyses addressed the influence of ROI geometry on the extracted values: within each joint, SUVmean was correlated across participants with the number of voxels in that joint’s ROI, and the between-subject variability and ROI-size dependence of SUVmean were compared with those of SUVmax. Three scans (two FDG, one NaF) returned non-physiological SUVs across all joints, indicating a failure of SUV normalization, and were excluded from these analyses. Unlike the correlation analyses, which were restricted to the healthy-volunteer subgroup, these geometric analyses were performed on the full CAMONA cohort in order to maximize the number of paired acquisitions available. The sample sizes reported for them, 109 participants with paired sessions and 110 to 125 scans per joint, therefore exceed those of the primary analyses.

### Statistical analysis

Pearson correlation coefficients were calculated to examine for associations between the SUVmean in the different weight-bearing joints and patient clinical factors including BMI and age. Statistical significance was set at *p* < 0.05. SUVmean values for each radiotracer, FDG and NaF, were analyzed separately across the 30 weight-bearing joints. Because each radiotracer–covariate combination generates 30 simultaneous tests, the Benjamini–Hochberg step-up procedure was applied to control the false discovery rate (FDR) within each of the four families (NaF–BMI, FDG–BMI, NaF–age and FDG–age). FDR-adjusted q values are reported alongside uncorrected p values in Tables 2, 3, 4 and 5, associations with q < 0.05 are regarded as significant, and associations reaching *p* < 0.05 but not q < 0.05 are identified in the tables and interpreted as exploratory. Pearson correlation estimates the linear component of each association; each joint region was modeled independently, and no higher-order age terms were fitted. Statistical analysis was calculated using the Python SciPy package.

## Results

The baseline clinical characteristics of the study are summarized in Table 1. A total of 89 healthy volunteers were eligible for inclusion; the number with a usable scan differed between the two tracers, as detailed below. There was a wide age range (21–75 years) with mean age 44.6 ± 13.9 years, and 52.8% of the population was male. The average BMI was 26.5 ± 4.4. A total of 76 scans using FDG and 80 scans using NaF were segmented; after exclusion of one FDG scan with failed SUV normalization, 75 FDG and 80 NaF scans entered the correlation analyses. Using AI-based segmentation, the average time spent per scan was 1.5 min to segment 30 joints, compared to 30 min for manual segmentation. Of the 62 associations reaching an uncorrected *p* < 0.05, nine did not survive Benjamini–Hochberg correction within their family; these are identified in Tables 2, 3, 4 and 5 and are flagged individually below. Because the procedure operates within families, the effective threshold differs between them: associations with p as high as 0.030 retained significance in the FDG–BMI family, whereas an association with *p* = 0.012 did not in the FDG–age family.

**Table 1:** Clinical Characteristics: The table summarizes demographic and clinical characteristics stratified by sex, including the total number of patients and descriptive statistics (mean ± SD, median, min, and max) for age and BMI

Statistic	Count	Age (years)	BMI
Male	47.00		
Female	42.00		
Total Patients	89.00		
Mean ± SD		44.58 ± 13.91	26.52 ± 4.41
Median		44.00	25.67
min		21.00	17.75
max		75.00	42.06

**Table 2:** NaF vs. BMI: Regions with significant correlations between BMI and NaF uptake. Pearson correlation coefficients (r) and corresponding *p*-values are shown for anatomical regions where a significant correlation (*p* < 0.05) was observed between BMI and NaF uptake. Benjamini–Hochberg FDR-adjusted q values are reported alongside the uncorrected *p* values; associations meeting *p* < 0.05 but not q < 0.05 are indicated

Joint	Pearson *r*	*p* value	BH-adjusted q
Intervertebral joint L5–S1	0.298	0.010	0.030
Intervertebral joint T10–T11	0.289	0.013	0.032
Intervertebral joint T9–T10	0.313	0.007	0.023
Intervertebral joint T7–T8	0.278	0.017	0.039
Intervertebral joint T6–T7	0.261	0.026	0.052†
Intervertebral joint T5–T6	0.349	0.003	0.015
Intervertebral joint T4–T5	0.341	0.003	0.015
Intervertebral joint T3–T4	0.292	0.012	0.033
Intervertebral joint C2–C3	0.262	0.025	0.054†
Right sacroiliac joint	0.464	< 0.001	0.007
Left sacroiliac joint	0.436	< 0.001	0.007
Right hip	0.321	0.004	0.017
Left hip	0.303	0.006	0.022
Right knee	0.605	< 0.001	0.007
Left knee	0.548	< 0.001	0.007

† Association meets the uncorrected threshold of *p* < 0.05 but not the Benjamini–Hochberg false discovery rate threshold of q < 0.05, and is interpreted as exploratory. q values were computed within a family of 30 simultaneous tests

**Table 3:** FDG vs. BMI: Regions with significant correlations between BMI and FDG uptake. Pearson correlation coefficients (r) and corresponding *p*-values are shown for anatomical regions where a significant correlation (*p* < 0.05) was observed between BMI and FDG uptake. Benjamini–Hochberg FDR-adjusted q values are reported alongside the uncorrected *p* values; associations meeting *p* < 0.05 but not q < 0.05 are indicated

Joint	Pearson *r*	*p* value	BH-adjusted q
Intervertebral joint L5–S1	0.523	< 0.001	0.002
Intervertebral joint L4–L5	0.616	< 0.001	0.002
Intervertebral joint L3–L4	0.434	< 0.001	0.002
Intervertebral joint L2–L3	0.277	0.019	0.025
Intervertebral joint L1–L2	0.466	< 0.001	0.002
Intervertebral joint T12–L1	0.484	< 0.001	0.002
Intervertebral joint T11–T12	0.353	0.002	0.004
Intervertebral joint T10–T11	0.340	0.003	0.005
Intervertebral joint T9–T10	0.348	0.003	0.005
Intervertebral joint T5–T6	0.322	0.006	0.009
Intervertebral joint T4–T5	0.382	< 0.001	0.002
Intervertebral joint T3–T4	0.521	< 0.001	0.002
Intervertebral joint T2–T3	0.427	< 0.001	0.002
Intervertebral joint T1–T2	0.235	0.047	0.054†
Intervertebral joint C7–T1	0.279	0.017	0.023
Intervertebral joint C6–C7	0.266	0.024	0.030
Intervertebral joint C5–C6	0.335	0.004	0.006
Intervertebral joint C4–C5	0.384	< 0.001	0.002
Intervertebral joint C3–C4	0.436	< 0.001	0.002
Intervertebral joint C2–C3	0.460	< 0.001	0.002
Intervertebral joint C1–C2	0.516	< 0.001	0.002
Right sacroiliac joint	0.597	< 0.001	0.002
Left sacroiliac joint	0.513	< 0.001	0.002
Left hip	0.248	0.030	0.036
Right knee	0.550	< 0.001	0.002
Left knee	0.518	< 0.001	0.002

† Association meets the uncorrected threshold of *p* < 0.05 but not the Benjamini–Hochberg false discovery rate threshold of q < 0.05, and is interpreted as exploratory. q values were computed within a family of 30 simultaneous tests

**Table 4:** NaF vs. Age: Regions with significant correlations between age and NaF uptake. Pearson correlation coefficients (r) and corresponding *p*-values are shown for anatomical regions where a significant correlation (*p* < 0.05) was observed between age and NaF uptake. Benjamini–Hochberg FDR-adjusted q values are reported alongside the uncorrected *p* values; associations meeting *p* < 0.05 but not q < 0.05 are indicated

Joint	Pearson *r*	*p* value	BH-adjusted q
Intervertebral joint L2–L3	−0.253	0.031	0.072†
Intervertebral joint L1–L2	−0.301	0.010	0.033
Intervertebral joint T12–L1	−0.232	0.048	0.103†
Intervertebral joint T11–T12	−0.272	0.020	0.055†
Intervertebral joint T7–T8	−0.270	0.021	0.052†
Intervertebral joint T6–T7	−0.316	0.007	0.026
Intervertebral joint T4–T5	−0.286	0.014	0.042
Intervertebral joint C6–C7	0.329	0.005	0.025
Intervertebral joint C5–C6	0.430	< 0.001	0.007
Intervertebral joint C4–C5	0.382	< 0.001	0.007
Intervertebral joint C3–C4	0.336	0.004	0.024
Intervertebral joint C1–C2	0.317	0.006	0.026
Right sacroiliac joint	−0.519	< 0.001	0.007
Left sacroiliac joint	−0.524	< 0.001	0.007

† Association meets the uncorrected threshold of *p* < 0.05 but not the Benjamini–Hochberg false discovery rate threshold of q < 0.05, and is interpreted as exploratory. q values were computed within a family of 30 simultaneous tests

**Table 5:** FDG vs. Age: Regions with significant correlations between age and FDG uptake. Pearson correlation coefficients (r) and corresponding *p*-values are shown for anatomical regions where a significant correlation (*p* < 0.05) was observed between age and FDG uptake. Benjamini–Hochberg FDR-adjusted q values are reported alongside the uncorrected *p* values; associations meeting *p* < 0.05 but not q < 0.05 are indicated

Joint	Pearson *r*	*p* value	BH-adjusted q
Intervertebral joint T4–T5	−0.243	0.040	0.171†
Intervertebral joint T3–T4	−0.294	0.012	0.060†
Intervertebral joint T2–T3	−0.331	0.004	0.030
Right sacroiliac joint	−0.350	0.002	0.030
Left sacroiliac joint	−0.336	0.003	0.030
Right knee	0.323	0.006	0.036
Left knee	0.371	0.002	0.030

† Association meets the uncorrected threshold of *p* < 0.05 but not the Benjamini–Hochberg false discovery rate threshold of q < 0.05, and is interpreted as exploratory. q values were computed within a family of 30 simultaneous tests

Paired FDG-session and NaF-session acquisitions were available for 109 participants, of whom 82 had the two sessions reconstructed onto a matching voxel grid. In the remaining 27, the per-participant median ratio of paired ROI volumes clustered near four or near one quarter and did so consistently across every joint, which indicates a twofold difference in linear voxel dimension between the two reconstructions and not a difference in segmentation, and these participants were excluded from the agreement analysis. Among the 82 participants with matching grids, joint ROI volumes derived from the two independent acquisitions agreed closely in every joint examined, with a median Pearson correlation of 0.98 (interquartile range 0.97–0.99; lowest value 0.91), a median intraclass correlation coefficient of 0.94 (interquartile range 0.93–0.96) and a median systematic difference of 0.6%. The median within-subject coefficient of variation was 13.6% (interquartile range 10.8–16.0%), corresponding to a 95% repeatability coefficient of + 46% and − 31% on the ratio scale, with the asymmetry arising because the coefficient of variation was estimated on the logarithmic scale. The pipeline therefore recovers the same joint volume from two separate acquisitions on average, although the difference between any individual pair of measurements is not negligible. Because the two sessions were acquired on separate days without a common spatial reference frame, spatial overlap between the paired ROIs could not be quantified, so this comparison addresses ROI volume alone (Supplementary Table S1).

The dependence of SUVmean on ROI size was negligible for FDG but regionally structured for NaF. For FDG the correlation between SUVmean and ROI voxel count had a median across joints of − 0.03, with no joint reaching statistical significance. For NaF the median was + 0.05, but this summary conceals a consistent regional pattern. All seven cervical intervertebral joints showed a positive correlation between SUVmean and ROI voxel count, from r = + 0.25 at C2-C3 to r = + 0.42 at C5-C6 (all *p* ≤ 0.005); these were the seven highest values in the series and the only ones exceeding r = + 0.13. Three lumbar joints showed weaker negative correlations (L1-L2, L2-L3 and L3-L4; *r* = − 0.20 to − 0.22, *p* < 0.03), and the remaining twenty joints showed no significant dependence in either direction. SUVmax behaved differently. It exceeded SUVmean by approximately twofold for both tracers, was more variable between participants than SUVmean for NaF in every joint examined (median between-subject coefficient of variation 44.6% versus 24.8%) and in 21 joints for FDG (63.1% versus 56.3%), and was considerably more dependent on ROI size, the median correlation with voxel count being + 0.51 for NaF and + 0.16 for FDG against + 0.05 and − 0.03 respectively for SUVmean (Supplementary Table S2).

**Fig. 6:**
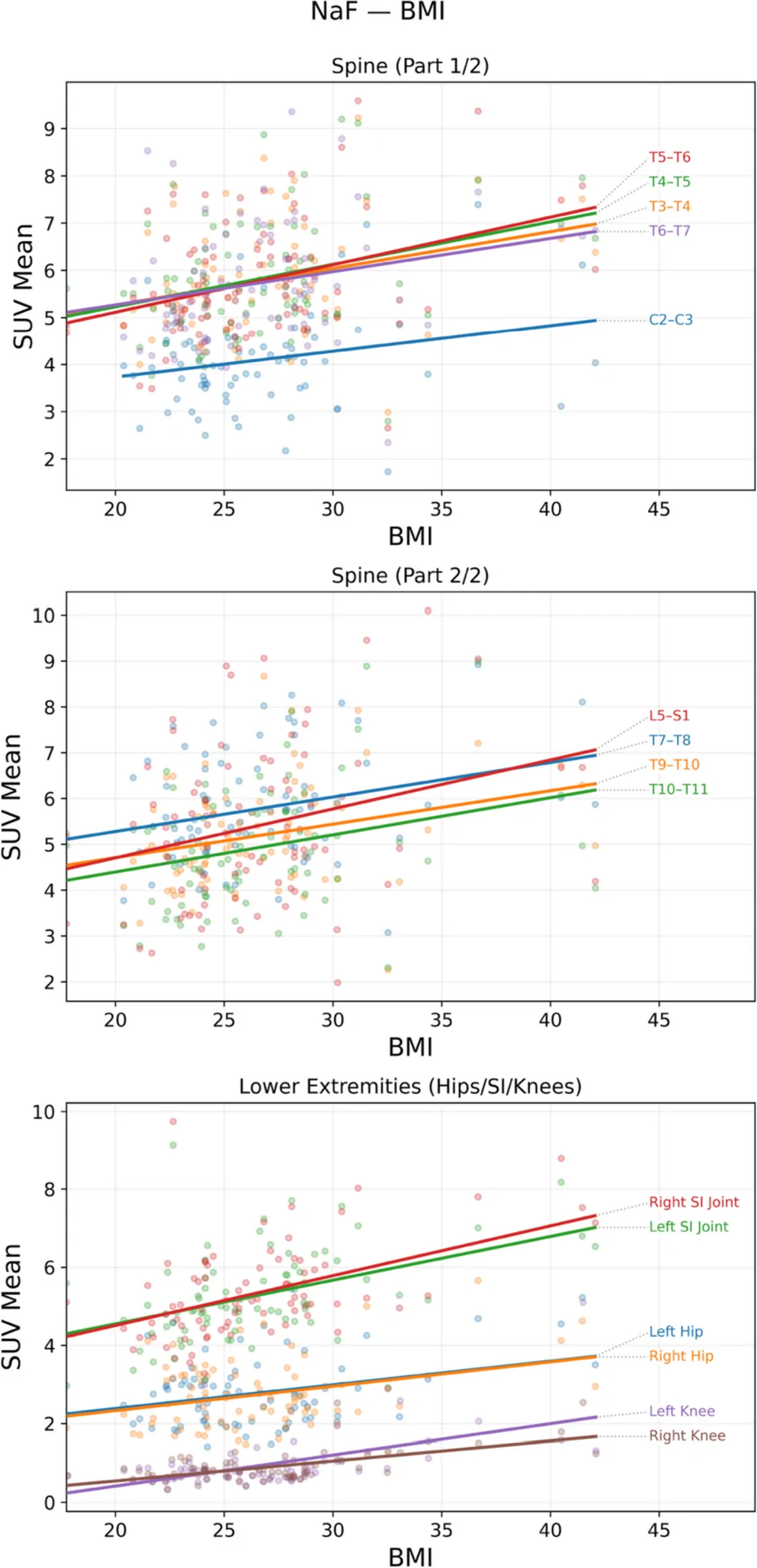
NaF vs. BMI Vertebral, Hip, SI, and Knee Regions: Positive correlation between BMI and NaF uptake across multiple joint and disk regions. Scatter plots show SUVs of NaF versus BMI for: vertebral IVDs, SI, hip, and knee joints. All regions demonstrated significant positive correlations between BMI and NaF uptake. For each significant quantified region, a linear regression trend line was overlaid on the scatter plots to represent the relationship between SUV and BMI

NaF SUVmean positively correlated with BMI in the joints of the lumbar (L5-S1) (*r* = 0.30, *p* = 0.01) and thoracic (T3-T6, T7-T8, T9-T11) (*r* = 0.30, *p* < 0.03) spine. The associations at T6-T7 (*r* = 0.26, *p* = 0.026) and at C2-C3 in the cervical spine (*r* = 0.26, *p* = 0.025) met the uncorrected threshold but not the corrected one and are reported as exploratory. The strongest associations were observed in the T5-T6 (*r* = 0.349, *p* < 0.003) and T4-T5 (*r* = 0.341, *p* = 0.003) joints. The SUVmean of the bilateral SI and hip joints positively correlated with BMI (*r* = 0.45, *p* < 0.001 and *r* = 0.31, *p* < 0.007, respectively) as well as both knees (*r* = 0.58, *p* < 0.001). All results are displayed in Table 2; Fig. 6.

**Fig. 7:**
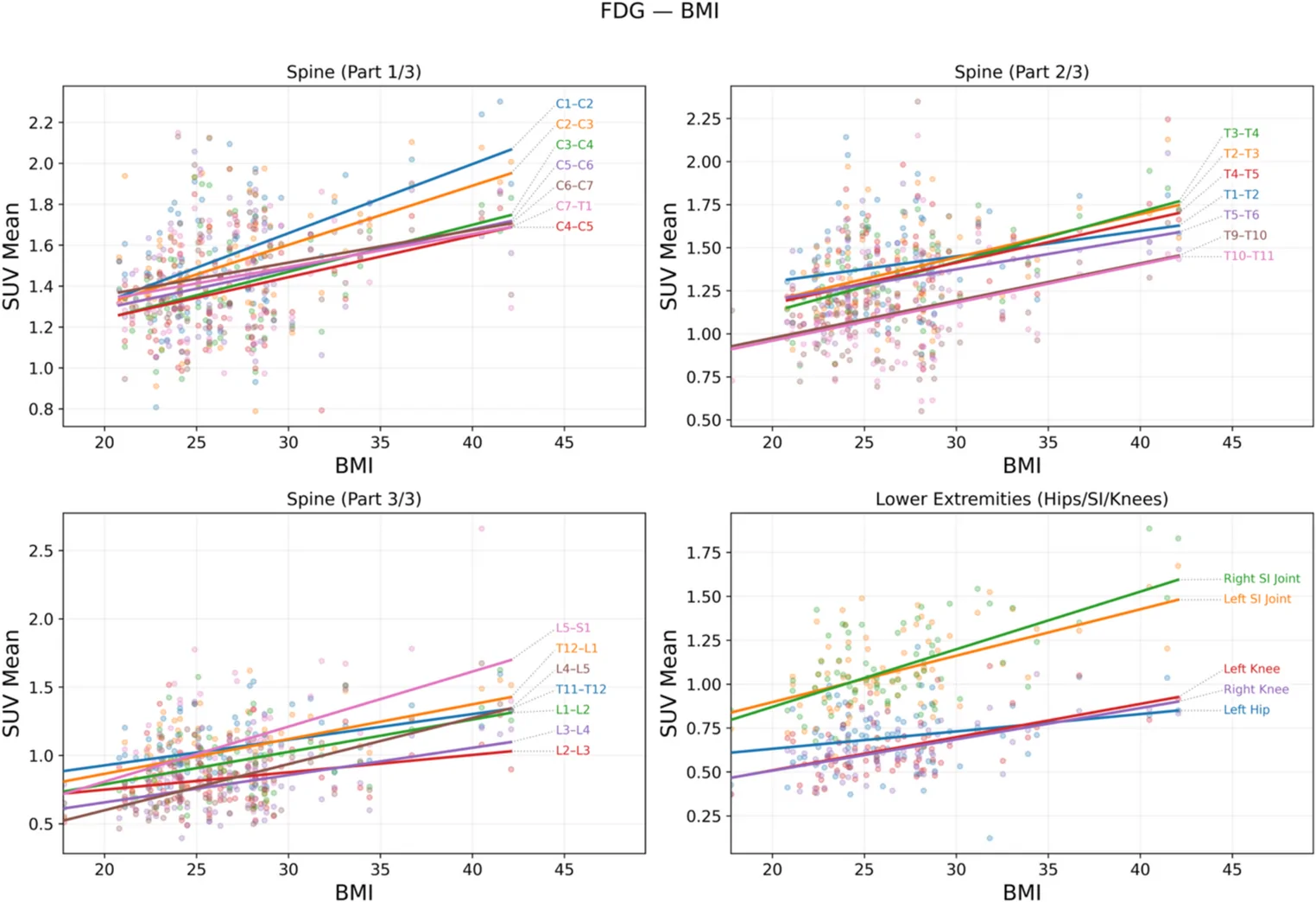
FDG vs. BMI Vertebral, Hip, SI, and Knee Regions: Positive correlation between BMI and FDG uptake across multiple joint and disk regions. Scatter plots show SUVs of FDG plotted against BMI for: vertebral IVDs, SI, hip, and knee joints. All charted regions showed significant positive correlations between BMI and FDG uptake. For each significant quantified region, a linear regression trend line was overlaid on the scatter plots to represent the relationship between SUV and BMI

FDG SUVmean positively correlated with BMI in all joints of C1-T6 and T9-S1, with the exception of T1-T2, where the association met the uncorrected threshold but not the corrected one (*p* = 0.047) and is reported as exploratory. Strongest correlations were found in the L4-L5 (*r* = 0.62, *p* < 0.001) and L5-S1 (*r* = 0.52, *p* < 0.001). Positive correlations were also observed in the bilateral SI (*r* = 0.56, *p* < 0.001), left hip (*r* = 0.25, *p* = 0.03), and bilateral knee joints (*r* = 0.53, *p* < 0.001). All results are described in Table 3; Fig. 7.

**Fig. 8:**
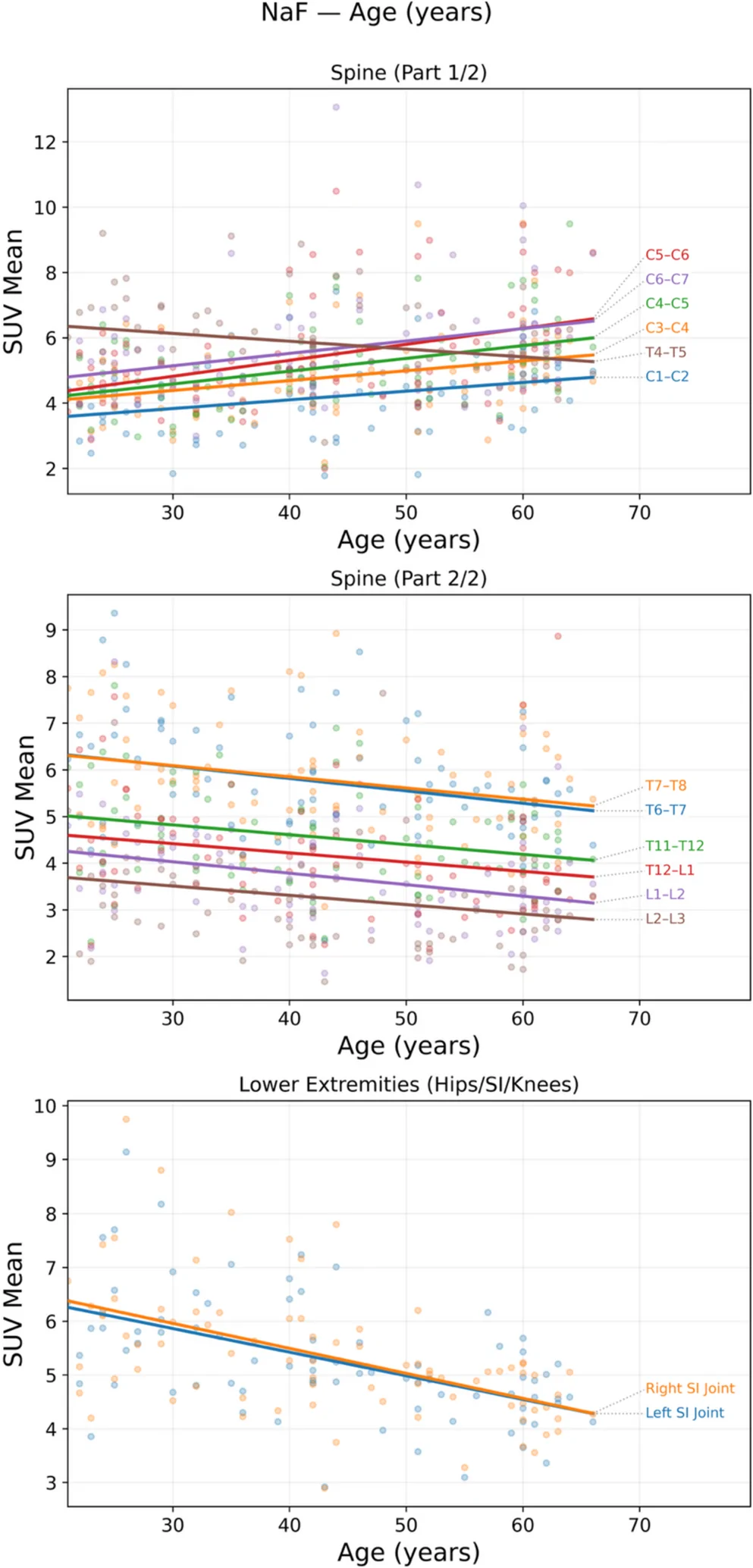
NaF vs. Age: Significant correlations between age and NaF uptake in vertebral IVDs and SI joints. Scatter plots show SUVs of NaF versus age for IVDs in the spine that were found to have significant positive or negative correlations. For each significant quantified region, a linear regression trend line was overlaid on the scatter plots to represent the relationship between SUV and age

Several associations were found between SUVmean and age for both FDG and NaF. NaF SUVmean positively correlated with age in the C1-C2 and C3-C7 joints (*r* = 0.36, *p* < 0.01). Significant negative correlations were found between NaF SUVmean and age at the T4-T5, T6-T7 and L1-L2 vertebral levels (*r* = -0.30, *p* ≤ 0.014) as well as the bilateral SI joints (*r* = -0.52, *p* < 0.001). The negative associations at T7-T8, T11-T12, T12-L1 and L2-L3 met the uncorrected threshold but not the corrected one and are reported as exploratory. These NaF results are described in Table 4; Fig. 8.

**Fig. 9:**
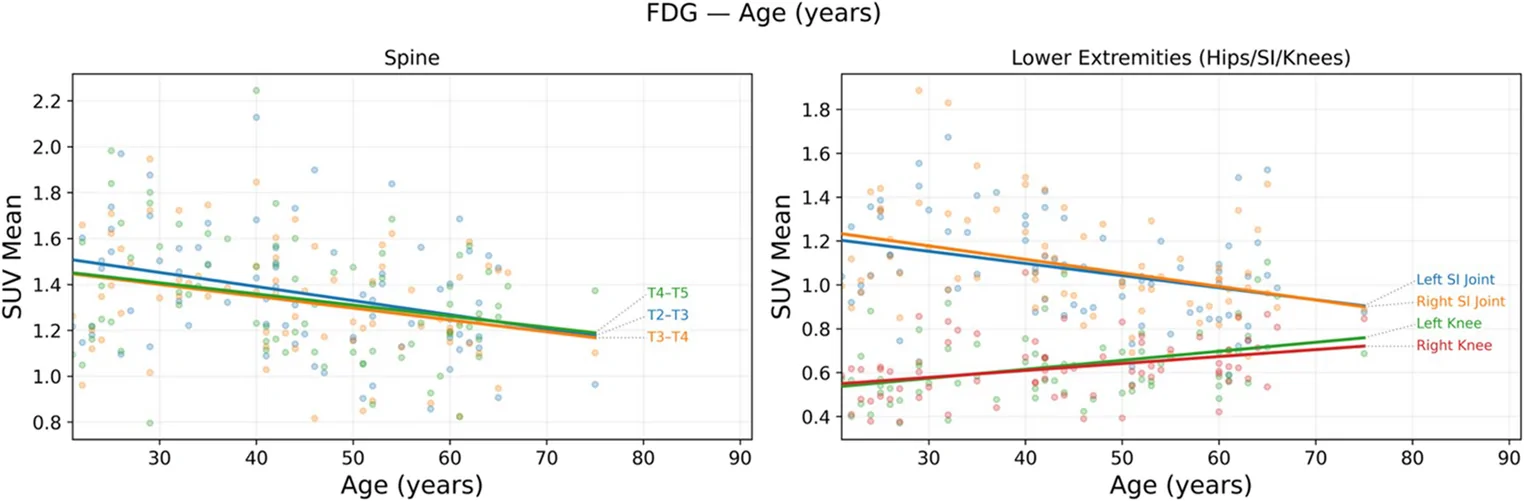
FDG vs. Age: Significant correlations between age and FDG uptake across the spine and lower extremities. SUVs of FDG are plotted against age for significant regions of the thoracic spine, knees, and bilateral SI joints. For each significant quantified region, a linear regression trend line was overlaid on the scatter plots to represent the relationship between SUV and age

FDG SUVmean positively correlated with age in the bilateral knee joints (*r* = 0.35, *p* < 0.01). A significant negative correlation was found between FDG uptake and age at the T2-T3 level (*r* = -0.33, *p* = 0.004) and in the bilateral SI joints (*r* = -0.34, *p* < 0.01); the negative associations at T3-T4 and T4-T5 did not survive correction and are reported as exploratory. These FDG results are described in Table 5; Fig. 9.

## Discussion

In this study, we developed and evaluated an AI-based method to automatically and reproducibly obtain quantitative measures of FDG and NaF PET uptake in several weight-bearing joint regions. We utilized combined PET/CT imaging from a cohort of healthy volunteers to obtain FDG and NaF SUVmean values in the 30 regions encompassing the IVDs, SI, hip, and knee joints. For each scan, the AI-based method generated values in about 1.5 min, a significant reduction in time compared to manual segmentation, which required around 30 min.

After FDR correction, NaF uptake in the IVDs of T3–T6, T7–T8, T9–T11 and L5–S1, as well as in the bilateral SI, hip and knee joints, was significantly positively correlated with BMI. NaF uptake also showed a significant positive correlation with age in the IVDs of C1-C2 and C3-C7, and significant negative correlations with age at T4-T5, T6-T7 and L1-L2 as well as in the bilateral SI joints. FDG uptake in every IVD between C1–T6 and T9–S1 other than T1-T2, as well as in the bilateral SI joints, left hip joint and knee joints, was significantly positively correlated with BMI. FDG uptake also showed a significant positive correlation with age in the bilateral knees. FDG also showed significant negative correlations with age at T2-T3 and in the bilateral SI joints. The nine borderline associations that met *p* < 0.05 but not q < 0.05 are identified in Tables 2, 3, 4 and 5.

To date, only a limited number of studies have investigated metabolic alterations in weight-bearing joints with PET, often utilizing a manual segmentation approach to obtain a ROI for radiotracer quantification [19–22]. A major strength of our study is the AI-based segmentation of the ROIs, which provides a more time-efficient and reproducible analytical approach for PET/CT. Our primary concern was whether the model captures SUVmean within the intended joint regions. Because the algorithm is deterministic, it produces identical segmentations for a given input scan and eliminates the interobserver variability inherent to manual ROI definition. Additionally, we evaluated the performance of our model across 30 major weight-bearing joints of the body for both FDG and NaF. The findings demonstrate the versatility of this algorithm to handle anatomical variations between different joint regions, as well as its ability to quantify signals for multiple PET radiotracers.

The findings of this study extend a growing body of evidence demonstrating the clinical relevance of FDG and NaF PET/CT in joint disease. The association of both radiotracers with weight-bearing degeneration (i.e., BMI) enables their use in detecting molecular alterations occurring in joint spaces prior to structural pathology or clinical symptoms [23]. The age-related increases in FDG uptake found in our study of healthy subjects have also been found in patients with osteoarthritis [24]. Both weight-bearing and aging can induce inflammatory changes in joint spaces, leading to a higher FDG signal [9, 25, 26]. The relationship of NaF uptake with age has been confirmed in other studies analyzing joints such as the shoulders, hip, and knee [27]. Alterations in NaF signal reflect the changes in activity of osteoblasts and osteoclasts within joint spaces that are susceptible to the deteriorating effects of weight-bearing and age [28, 29].

Interestingly, while some regions showed increased uptake with age suggesting ongoing metabolic or inflammatory processes, other regions showed significantly decreased tracer activity, potentially reflecting later-stage degenerative changes. These findings hint at the complex region and tracer-specific nature of joint degeneration. While increased uptake in regions such as the cervical spine and knees suggests active inflammatory or remodeling processes consistent with early degeneration, subject for the cervical spine to the geometric caveat, significant negative correlations observed in areas such as the mid-thoracic spine and SI joints can indicate more advanced degenerative changes characterized by reduced cellular metabolic activity. For instance, the declining NaF signal at T4-T5 and T6-T7 may suggest diminished osteoblastic activity as these regions transition from active remodeling to sclerosis or fibrosis, while lower FDG signal may suggest resolution of inflammatory processes in favor of structural stabilization. These findings can be supported by prior research showing that NaF uptake declines in sclerotic lesions due to reduced osteoblastic activity, and that FDG signal decreases in regions of chronic inflammation as they evolve into fibrotic, metabolically inactive tissue [30, 31]. Another explanation for these results could be due to decreased vascular perfusion with age leading to lower NaF delivery to these joints, further contributing to regional decreases in signal [32]. This account describes heterogeneity between anatomical regions in a cross-sectional cohort, not a trajectory within any single joint; a biphasic within-region course is a plausible extension but cannot be established from these data.

Our pipeline pairs a deep learning bone segmentation with a deterministic, distance-based rule for defining the intervening joint space, and it is worth contrasting this hybrid design with a dedicated end-to-end network trained directly on joint boundaries, such as a 3D U-Net [33]. The geometric approach requires no joint-specific annotation, extends to any pair of adjacent bones that TotalSegmentator recognizes, and remains interpretable, because the ROI is an explicit function of the bone masks and a single distance parameter. Its principal weakness is that a fixed distance rule presumes a measurable gap between adjacent bone surfaces. That assumption fails in advanced degenerative disease with bone-on-bone contact, subchondral sclerosis, marginal osteophytes or bridging syndesmophytes, where the ROI may shrink below the appearance threshold and be discarded, or may be displaced into dense sclerotic bone and inflate the NaF signal. A network trained on expert-delineated joint capsules would learn the appearance of the joint space itself instead of inferring it from bone geometry, and would in principle remain valid where the space is obliterated [15, 34]. That gain carries costs, because annotation across 30 dissimilar sites is expensive and reintroduces the interobserver variability this method eliminates, and the training set would have to over-represent pathological joints. We intend to retain the current pipeline for population-scale analysis while developing a supervised refinement stage for degenerative joints, initialized from the geometric masks. In the present cohort, the risk of sclerotic contamination is mitigated, though not eliminated, by the exclusion of participants with major skeletal deformity and by the discarding of any joint ROI smaller than 50 voxels.

The joint spaces analyzed here are thin relative to the reconstructed spatial resolution of clinical PET, so every joint measurement is affected by partial volume effects, with spill-out from the joint space and spill-in from the adjacent, intensely NaF-avid endplates and subchondral bone [35]. SUVmean was chosen deliberately in preference to SUVmax. In a structure only a few voxels thick and bounded on both sides by bone that concentrates NaF far more avidly than the intervening tissue, SUVmax is determined by the single hottest voxel within the ROI, which in practice lies at the bone interface; SUVmax would therefore be more, not less, sensitive to spill-in from bone, in addition to being noise-dependent and poorly comparable across scanners and reconstruction settings. The sensitivity analysis supports this. SUVmax was approximately twice SUVmean, was the more variable of the two between participants, and was several times more strongly correlated with ROI size, which is the geometric dependence that a partial volume artifact would be expected to introduce. SUVpeak is similarly ill-defined, because the joint ROI is a thin curved sheet two to three voxels thick and cannot accommodate the conventional 1 cm³ sphere at any total volume. Substituting either metric would therefore make the measurement more susceptible to the confounder that motivated the suggestion.

Observed age and BMI associations might themselves be partly geometric. First, SUVmean showed no material dependence on ROI voxel count for FDG at any joint, and none for NaF outside the cervical spine, so for most joints no systematic relationship between the size of the segmented joint space and the extracted value is detectable in these data. The cervical joints are the exception. All seven showed a positive correlation between NaF SUVmean and ROI voxel count, from r = + 0.25 at C2-C3 to r = + 0.42 at C5-C6, and these were the only joints in which the correlation exceeded r = + 0.13. Because the positive NaF–age associations are confined to these same joints, a geometric contribution to that finding cannot be excluded. Every other association, including all of the BMI associations and all of the negative age associations, arises in joints where no such dependence is detectable. Second, progressive joint-space narrowing would increase bone spill-in and bias all associations positively, yet significant negative age correlations were observed for both tracers in the mid-thoracic and sacroiliac joints. Third, the pattern of significant associations differs between the two tracers within the same anatomical regions, with positive age associations confined to the cervical intervertebral joints for NaF and to the knees for FDG, whereas a purely volumetric artifact acting through ROI size alone would affect both tracers comparably in the same geometry. This dissociation is weaker evidence than it first appears, because the dependence of SUVmean on ROI size is itself tracer-specific and is present for NaF but not FDG in the cervical spine, so it cannot be treated as independent evidence against a geometric contribution at those joints.

Reproducibility of an automated quantification tool has two separable components [36]. The segmentation step is deterministic, so the interobserver and intraobserver variance that dominates manual ROI placement is zero by construction, although this does not address the variance introduced upstream by repositioning, respiration and reconstruction differences. No same-tracer scan–rescan acquisition was part of the CAMONA protocol, but the two-tracer design permits a partial assessment, each participant having undergone two sessions on different days with independent positioning and wholly independent runs of the pipeline. Agreement was high, with a median Pearson correlation across joints of 0.98, a median intraclass correlation coefficient of 0.94 and a median systematic difference below 1%, which indicates that the geometric step of the method is robust to ordinary acquisition-to-acquisition variation in positioning and reconstruction. The median within-subject coefficient of variation of 13.6%, corresponding to a repeatability coefficient of + 46% and − 31% on the ratio scale, is more relevant to longitudinal use, because it sets a floor on the change a single measurement could resolve. The analysis addresses ROI volume alone, and both ROI position and the reproducibility of SUV itself remain unquantified, the latter because the two sessions used different tracers. A prospective same-tracer scan–rescan substudy is still required before the tool is used to track change within an individual patient.

The runtime reported here was achieved on a single consumer-grade workstation graphics card, with no dedicated server or cluster hardware. This matters for departments considering deployment, because the marginal hardware cost is that of a desktop GPU and comparable performance is available from any current CUDA-capable card with sufficient video memory. Cost is therefore unlikely to be the binding constraint for most academic nuclear medicine departments, and restrictions on installing GPU hardware within a clinical network and on moving images outside the PACS environment are often more limiting than the price of the card. CPU-only execution remains possible, since TotalSegmentator supports it directly [37], but the penalty is substantial: published runtime documentation reports full-resolution inference of the order of 40 to 50 min per study on CPU, against well under a minute on a CUDA-capable GPU, with the reduced-resolution option bringing CPU runtime down to approximately a minute at the cost of segmentation detail [38]. A CPU-only deployment would therefore erase most of the per-study efficiency advantage over manual segmentation, although it would remain viable for overnight batch processing of research cohorts, where the primary benefit is operator independence and not turnaround time.

Regarding statistical treatment, the analysis comprises 30 tests for each tracer–covariate combination, and the original submission reported uncorrected *p* values throughout. Applying the Benjamini–Hochberg procedure within each family [39] leaves the direction and the anatomical distribution of the principal findings unchanged, although several borderline associations no longer meet the corrected threshold and the extent of some regional clusters is correspondingly narrower. These are identified in Tables 2, 3, 4 and 5 and are interpreted as exploratory. Pearson correlation also estimates only the linear component of each relationship, which suits the region-wise, cross-sectional associations reported here but does not test the biphasic model that the regional pattern suggests. Distinguishing true non-linearity from between-region heterogeneity would require longitudinal imaging of the same joints, which is not possible in this dataset.

There are several limitations to our study that warrant consideration. First, data were analyzed retrospectively, which may introduce selection bias. Future studies can alleviate this limitation by implementing prospective design. Second, PET has relatively low spatial resolution, which may complicate AI-based quantification methods if there is significant radiotracer uptake in tissues adjacent to the target region. This is particularly relevant for the IVDs, as the neighboring vertebral bodies exhibit a high NaF signal. Formal partial volume correction was not applied and SUVmean was not adjusted for ROI volume, so a geometric contribution to the associations reported here cannot be excluded. This applies with particular force to the positive NaF–age associations in the cervical spine, which arise in the only joints where SUVmean was itself appreciably correlated with ROI voxel count. Third, we did not validate the method on an external dataset; although CAMONA participants were scanned on different PET/CT scanners, external validation remains necessary. Fourth, no same-tracer scan–rescan acquisitions were available, so only the consistency of the ROI-generation step could be established and the repeatability of the derived SUV values could not be quantified. Fifth, geometric accuracy was assessed against an intensity-based bone mask and not against expert manual delineations of the joint spaces, so Dice similarity coefficients and Hausdorff distances relative to a manually traced reference standard are not available. Sixth, the joint-space rule is parameterized in voxels and not in millimeters, so the physical dimensions of each ROI depend on the reconstructed voxel size of the CT series. The paired-session analysis indicated a twofold difference in linear voxel dimension between the two reconstructions in roughly a quarter of participants, which implies that the ROIs are not physically identical across the cohort; restating the thresholds in millimeters and resampling all series onto a common grid would remove this source of variation. Seventh, the cohort was recruited as healthy volunteers but was not screened for asymptomatic degenerative disease, so the values reported here describe an unselected ambulatory population and not a radiographically normal one. Finally, although many statistically significant correlations were found, there was considerable scatter in uptake, reflecting individual differences. Because of this wide variability, these correlations cannot provide precise insight into metabolic behavior at the individual joint level. Nevertheless, the relationship between NaF and FDG uptake in joints remains important for understanding disease mechanisms, informing treatment guidelines, and allowing for assessment of individual patients.

This study presents an automated joint segmentation technique using the TotalSegmentator model to quantify metabolic activity of joints with FDG and NaF PET/CT imaging. This method enables rapid, operator-independent PET/CT quantification relative to standard manual segmentation techniques. The efficiency and ease of use make this a promising tool for further research and supports the application of PET/CT in the detection of metabolic changes in joints.

## Supplementary Information

Below is the link to the electronic supplementary material.

Supplementary Material 1

## Data Availability

The datasets generated during and/or analyzed during the current study are available from the corresponding author, Abass Alavi, on reasonable request.
