# Supplementary material for: Automated joint segmentation enables quantitative molecular imaging of degeneration with [¹⁸F]FDG and [¹⁸F]NaF PET/CT
Source: Eur J Nucl Med Mol Imaging. 2026 Aug 21;53(13):7715–28. doi: 10.1007/s00259-026-08139-z (PMC13727869; doi:10.1007/s00259-026-08139-z)
Supplement: Supplementary file 1 [file 259_2026_8139_MOESM1_ESM.docx]

**Supplementary Tables**

**Supplementary Table S1. Agreement of automatically generated joint ROI volumes between the two independent PET/CT sessions of the same participant.**

*Restricted to the 82 participants whose two sessions were reconstructed onto a matching voxel grid. ICC, intraclass correlation coefficient; CV, coefficient of variation. Bias is the median systematic difference of the FDG-session volume relative to the NaF-session volume.*

| **Joint** | **n** | **Median voxels (FDG)** | **Median voxels (NaF)** | **Pearson r** | **ICC** | **Within-subject CV (%)** | **Bias (%)** |
| --- | --- | --- | --- | --- | --- | --- | --- |
| L5–S1 | 82 | 68 | 66 | 0.978 | 0.934 | 15.4 | +1.8 |
| L4–L5 | 82 | 104 | 99 | 0.972 | 0.964 | 11.3 | +1.7 |
| L3–L4 | 82 | 114 | 107 | 0.972 | 0.967 | 10.7 | +3.7 |
| L2–L3 | 82 | 104 | 109 | 0.935 | 0.931 | 15.8 | +0.2 |
| L1–L2 | 82 | 90 | 94 | 0.993 | 0.972 | 9.9 | -3.8 |
| T12–L1 | 82 | 78 | 78 | 0.985 | 0.976 | 9.1 | +1.9 |
| T11–T12 | 82 | 72 | 72 | 0.979 | 0.972 | 9.7 | +1.2 |
| T10–T11 | 82 | 63 | 67 | 0.974 | 0.968 | 10.8 | -0.5 |
| T9–T10 | 82 | 63 | 63 | 0.976 | 0.934 | 15.4 | +3.1 |
| T8–T9 | 82 | 59 | 58 | 0.955 | 0.928 | 16.8 | -2.5 |
| T7–T8 | 82 | 57 | 58 | 0.933 | 0.857 | 22.7 | -5.6 |
| T6–T7 | 82 | 53 | 52 | 0.986 | 0.955 | 12.4 | +1.5 |
| T5–T6 | 81 | 50 | 47 | 0.986 | 0.952 | 12.4 | +3.8 |
| T4–T5 | 81 | 45 | 42 | 0.976 | 0.950 | 12.5 | +1.1 |
| T3–T4 | 81 | 36 | 36 | 0.979 | 0.944 | 13.8 | -2.9 |
| T2–T3 | 81 | 30 | 30 | 0.982 | 0.945 | 13.5 | +1.3 |
| T1–T2 | 81 | 28 | 26 | 0.906 | 0.936 | 16.3 | +2.2 |
| C7–T1 | 81 | 26 | 25 | 0.972 | 0.928 | 17.0 | +3.4 |
| C6–C7 | 81 | 39 | 35 | 0.972 | 0.934 | 16.9 | +2.0 |
| C5–C6 | 81 | 46 | 45 | 0.987 | 0.959 | 13.2 | -0.8 |
| C4–C5 | 81 | 45 | 42 | 0.981 | 0.934 | 16.1 | -0.2 |
| C3–C4 | 81 | 38 | 38 | 0.979 | 0.933 | 15.5 | +0.8 |
| C2–C3 | 80 | 34 | 36 | 0.970 | 0.934 | 16.1 | -3.1 |
| C1–C2 | 78 | 50 | 46 | 0.972 | 0.944 | 13.8 | +1.4 |
| Left hip | 82 | 183 | 188 | 0.995 | 0.991 | 5.0 | -0.9 |
| Right hip | 82 | 175 | 175 | 0.997 | 0.993 | 4.4 | +0.5 |
| Left SI | 82 | 110 | 106 | 0.981 | 0.959 | 10.8 | +0.3 |
| Right SI | 82 | 108 | 112 | 0.986 | 0.959 | 10.4 | -1.6 |
| Left knee | 70 | 200 | 200 | 0.989 | 0.930 | 15.3 | -2.9 |
| Right knee | 70 | 198 | 191 | 0.986 | 0.897 | 18.7 | -2.8 |

**Supplementary Table S2. Dependence of SUVmean and SUVmax on ROI size, and between-subject variability of each metric.**

*Correlations are computed across participants within each joint, between the metric and the number of voxels in that joint’s ROI. CV, between-subject coefficient of variation.*

| **Joint** | **Tracer** | **n** | **r (SUVmean vs ROI voxels)** | **r (SUVmax vs ROI voxels)** | **CV SUVmean (%)** | **CV SUVmax (%)** |
| --- | --- | --- | --- | --- | --- | --- |
| L5–S1 | FDG | 119 | -0.121 | +0.100 | 74.9 | 78.4 |
| L5–S1 | NaF | 125 | +0.127 | +0.431 | 41.1 | 57.4 |
| L4–L5 | FDG | 119 | -0.130 | +0.133 | 105.4 | 82.1 |
| L4–L5 | NaF | 125 | -0.003 | +0.508 | 41.2 | 55.6 |
| L3–L4 | FDG | 119 | -0.126 | +0.123 | 99.4 | 76.5 |
| L3–L4 | NaF | 125 | -0.221 | +0.338 | 45.4 | 48.9 |
| L2–L3 | FDG | 119 | -0.037 | +0.224 | 88.4 | 81.5 |
| L2–L3 | NaF | 125 | -0.198 | +0.404 | 37.5 | 51.9 |
| L1–L2 | FDG | 119 | -0.108 | +0.134 | 77.2 | 73.5 |
| L1–L2 | NaF | 125 | -0.209 | +0.502 | 32.2 | 55.0 |
| T12–L1 | FDG | 119 | -0.085 | +0.070 | 73.4 | 66.7 |
| T12–L1 | NaF | 125 | -0.086 | +0.583 | 29.4 | 59.2 |
| T11–T12 | FDG | 119 | -0.069 | +0.136 | 67.1 | 72.6 |
| T11–T12 | NaF | 125 | -0.155 | +0.498 | 24.5 | 34.6 |
| T10–T11 | FDG | 119 | -0.011 | +0.185 | 69.8 | 81.1 |
| T10–T11 | NaF | 125 | +0.006 | +0.491 | 27.3 | 41.0 |
| T9–T10 | FDG | 119 | +0.008 | +0.180 | 58.7 | 69.4 |
| T9–T10 | NaF | 125 | -0.069 | +0.457 | 22.4 | 35.9 |
| T8–T9 | FDG | 119 | +0.004 | +0.201 | 65.4 | 71.3 |
| T8–T9 | NaF | 125 | +0.030 | +0.380 | 21.9 | 43.3 |
| T7–T8 | FDG | 119 | +0.014 | +0.197 | 61.4 | 66.2 |
| T7–T8 | NaF | 125 | +0.073 | +0.451 | 21.3 | 37.7 |
| T6–T7 | FDG | 119 | +0.004 | +0.241 | 60.4 | 58.7 |
| T6–T7 | NaF | 125 | -0.050 | +0.514 | 22.6 | 44.7 |
| T5–T6 | FDG | 118 | -0.015 | +0.190 | 54.0 | 57.5 |
| T5–T6 | NaF | 125 | +0.039 | +0.642 | 21.3 | 38.4 |
| T4–T5 | FDG | 118 | +0.043 | +0.235 | 43.6 | 52.8 |
| T4–T5 | NaF | 125 | +0.019 | +0.620 | 20.1 | 30.6 |
| T3–T4 | FDG | 118 | -0.019 | +0.139 | 50.6 | 59.3 |
| T3–T4 | NaF | 125 | +0.065 | +0.538 | 19.2 | 30.4 |
| T2–T3 | FDG | 118 | -0.046 | +0.086 | 53.8 | 66.7 |
| T2–T3 | NaF | 125 | +0.107 | +0.402 | 21.0 | 46.0 |
| T1–T2 | FDG | 118 | -0.039 | +0.152 | 51.1 | 54.2 |
| T1–T2 | NaF | 125 | +0.024 | +0.515 | 20.4 | 36.4 |
| C7–T1 | FDG | 118 | -0.030 | +0.233 | 40.5 | 45.7 |
| C7–T1 | NaF | 125 | +0.379 | +0.713 | 22.7 | 51.0 |
| C6–C7 | FDG | 118 | -0.015 | +0.255 | 42.0 | 54.7 |
| C6–C7 | NaF | 125 | +0.286 | +0.629 | 29.4 | 53.6 |
| C5–C6 | FDG | 117 | -0.046 | +0.200 | 37.6 | 50.4 |
| C5–C6 | NaF | 125 | +0.423 | +0.657 | 28.1 | 52.6 |
| C4–C5 | FDG | 117 | -0.023 | +0.154 | 45.4 | 58.5 |
| C4–C5 | NaF | 125 | +0.289 | +0.596 | 24.7 | 45.5 |
| C3–C4 | FDG | 117 | -0.002 | +0.215 | 34.4 | 65.6 |
| C3–C4 | NaF | 125 | +0.365 | +0.610 | 24.8 | 44.6 |
| C2–C3 | FDG | 116 | -0.065 | +0.148 | 38.0 | 66.6 |
| C2–C3 | NaF | 121 | +0.254 | +0.642 | 23.9 | 34.4 |
| C1–C2 | FDG | 113 | +0.013 | +0.181 | 26.1 | 47.3 |
| C1–C2 | NaF | 116 | +0.328 | +0.677 | 26.6 | 34.5 |
| Left hip | FDG | 119 | +0.005 | +0.191 | 61.2 | 58.6 |
| Left hip | NaF | 125 | +0.117 | +0.605 | 32.5 | 44.1 |
| Right hip | FDG | 119 | -0.021 | +0.143 | 79.7 | 66.2 |
| Right hip | NaF | 125 | +0.108 | +0.415 | 37.8 | 45.5 |
| Left SI | FDG | 119 | -0.083 | +0.085 | 64.0 | 60.7 |
| Left SI | NaF | 125 | -0.033 | +0.370 | 23.5 | 40.2 |
| Right SI | FDG | 119 | -0.047 | +0.169 | 44.2 | 48.9 |
| Right SI | NaF | 125 | -0.025 | +0.388 | 24.5 | 38.1 |
| Left knee | FDG | 110 | -0.134 | +0.084 | 28.3 | 49.6 |
| Left knee | NaF | 119 | +0.091 | +0.296 | 69.9 | 104.0 |
| Right knee | FDG | 110 | -0.101 | +0.125 | 35.0 | 51.0 |
| Right knee | NaF | 119 | +0.115 | +0.336 | 55.0 | 110.1 |
